# Supplementary material for: PRRSV Non-Structural Proteins Orchestrate Porcine E3 Ubiquitin Ligase RNF122 to Promote PRRSV Proliferation
Source: Viruses. 2022 Feb 18;14(2):424. doi: 10.3390/v14020424 (PMC8874583; doi:10.3390/v14020424)
Supplement: Supplementary file 1 [file viruses-14-00424-s001.zip › viruses-1588336-supplementary.pdf]

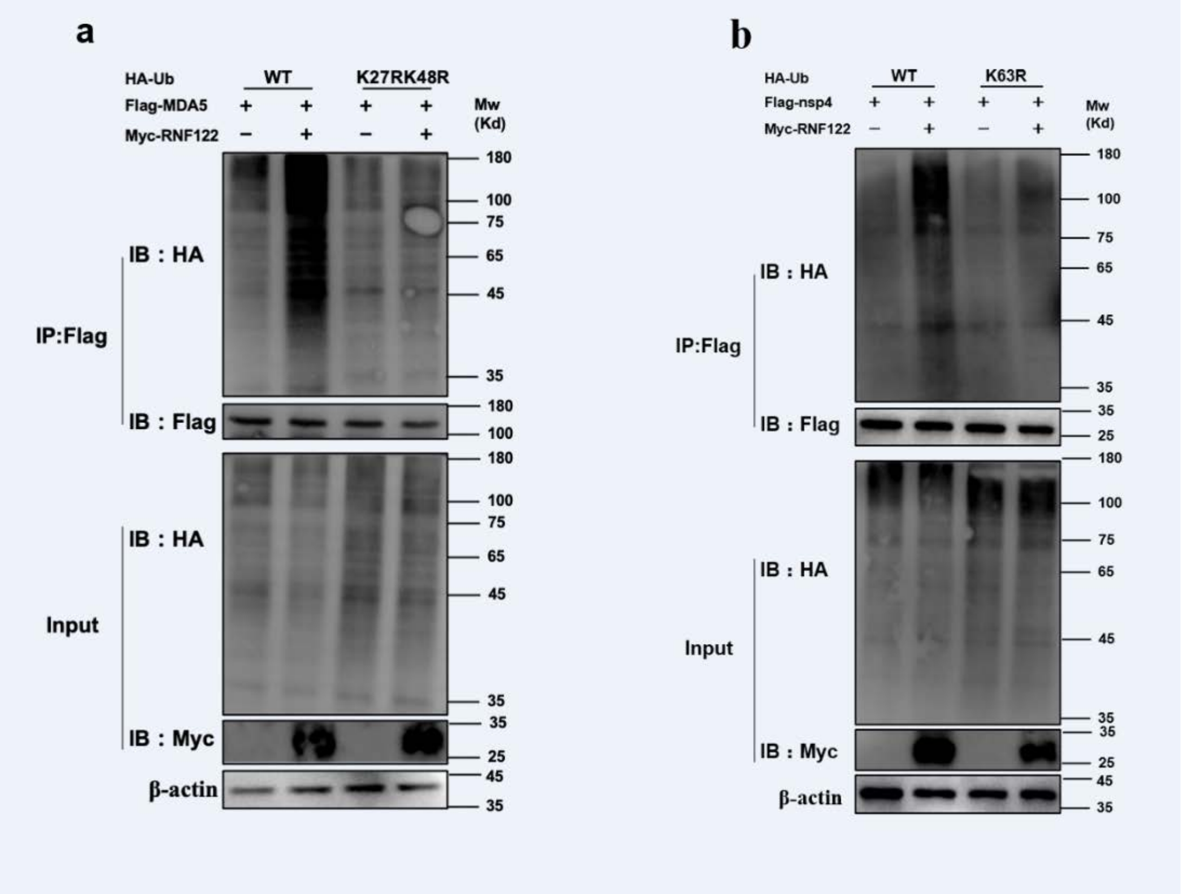

Figure S1. Porcine RNF122 performed K27-linked and K48-linked ubiquitination to MDA5 and medicate K63-linked ubiquitination to nsp4. （a）HEK293T cells were co-transfected with Myc-RNF122, Flag-MDA5 and HA-Ub (WT), HA-Ub (K27RK48R). 24 h after transfection, Flag Beads were used for CO-IP, and further detected by Western blotting with an anti-HA, an anti-Flag antibody and an anti-Myc antibody, respectively. （b）HEK293T cells were co-transfected with Myc-RNF122, Flag-nsp4 and HA-Ub (WT), HA-Ub (K63R). 24 h after transfection, Flag Beads were used for CO-IP, and further detected by Western blotting with an anti-HA, an anti-Flag antibody and an anti-Myc antibody, respectively.
